# Supplementary material for: Chromothripsis during telomere crisis is independent of NHEJ, and consistent with a replicative origin
Source: Genome Res. 2019 May;29(5):737–49. doi: 10.1101/gr.240705.118 (PMC6499312; doi:10.1101/gr.240705.118)
Supplement: Supplemental Material [file supp_gr.240705.118_Supplemental_file_1.zip › contigs/annotated_contigs/DB115/contig.2.DB115_length_819_mean_cov_31.5653235653.docx]

**DB115_length_819_mean_cov_31.5653235653**

```
CCT|TTCTCTTTTTCTCCCTCTTCCCCTCTCTCTCT|GCCTGTGCCTGTTCTCTCTC|TCTCTCTCTCTCTCTCTCTCTCTCTCTGTCT
    >chr3:89661231-89661263 + E=2e-01 p=0e+00           >chrUn_gl000220:128991-129761
CTCTCTCTCTCTCTCTCTCTCTCTCTCTCTCTCTCTCTCTGCCTTTCTCTCTCACTGTGTCGGTCTTCTGTCTTACTCTCTTTCTCTGC
+ E=0e+00
CTGCCTCTCTGTCTGTCTGTCTGTCTCTCTCCCTCCATGTCTCTCTCTCTCTCTCTCACTCACTCTCTCTCCGTCTCTCTCTCTTTCTG

TCTGTTTCTCTCTGTCTCTGTCTTTCTGTGTGTCTGTCTGTCTCTCTCTCTATTTGTCTTTCTCCCTCCCTGTCTGTTTCTCTCTCTCT

CTCTCTCTCTCTCTCTCTCTGTCTGTCTGTTTCTCTCTATCTCTCGCTGTCCATCTCTGTCTTTCTATGTCTGTCTCTTTCTCTGTCAG

TCTGTCAGACACCCCCGTGCCGGGTAGGGCCCTGCCCCTTCCACGAAAGTGAGAAGCGCGTGCTTCGGTGCTTAGAGAGGCCGAGAGGA

ATCTAGACAGGCGGGCCTTGCTGGGCTTCCCCACTCGGTGTATGATTTCGGGAGGTCGAGGCCGGGTCCCCGCTTGGATGCGAGGGGCA

TTTTCAGACTTTTCTCTCGGTCACGTGTGGCGTCCGTACTTCTCCTATTTCCCCGATAAGCTCCTCGACTTCAACATAAACGGCGTCCT

AAGGGTCGATTTAGTGTCATGCCTCTTTCACCGCCACCACCGAAGATGAAAGCAAAGATCGGCTAAATACCGCGTGTTCTCATCTAGAA

GTGGGAACTTACAGATGACAG
```
